# An Easily Neglected Rapid Progressive Emphysematous Liver Abscess

**DOI:** 10.14309/crj.0000000000000631

**Published:** 2021-07-13

**Authors:** Yu-Chi Tsai, Wen-Ching Kung, Chien-Wei Huang

**Affiliations:** 1 Department of Internal Medicine, Kaohsiung Armed Forces General Hospital, Kaohsiung, Taiwan; 2 Department of Gastroenterology and General Surgical Medicine, Kaohsiung Armed Forces General Hospital, Kaohsiung, Taiwan; 3 Department of Nursing, Tajen University, Taipei, Taiwan

## CASE REPORT

A 62-year-old woman with type 2 diabetes mellitus presented with increasing diffuse abdominal pain for 1 week. The patient denied smoking or alcohol use. The patient had been self-administering a Chinese herb to try to control her diabetes, but her glycemic control has been poor for the past 6 months. No other systemic disorder such as malignancy or hepatobiliary disease was recorded. Physical examination found ill appearance with diffuse abdominal tenderness. Her vital signs were a body temperature of 36.2 °C, blood pressure of 106/58 mm Hg, and heart rate of 116 bpm. The blood test showed white blood cell count of 13,190/μL with anemia (32.7%), C-reactive protein level of 27.47 mg/dL, and procalcitonin level of 32.23 ng/mL. Hyperglycemia with a glycated hemoglobin level of 11.0% was documented.

**Figure 1.**
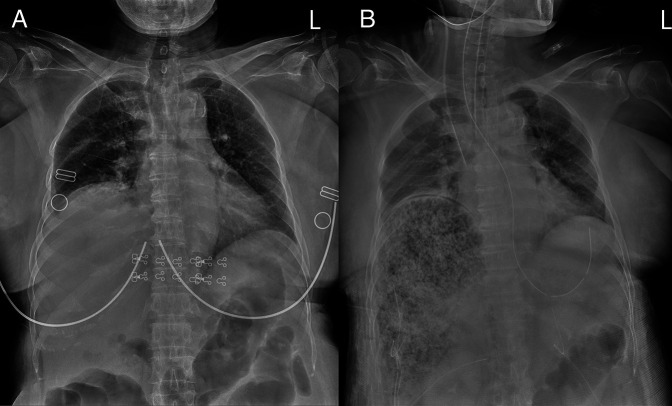
(A) Initial radiography demonstrated no prominent evidence of ongoing liver illness but showed a marked elevation of right-side diaphragm and ileus. (B) Two hours later, radiography showed gas-containing liver, with clinical rapid deterioration.

**Figure 2.**
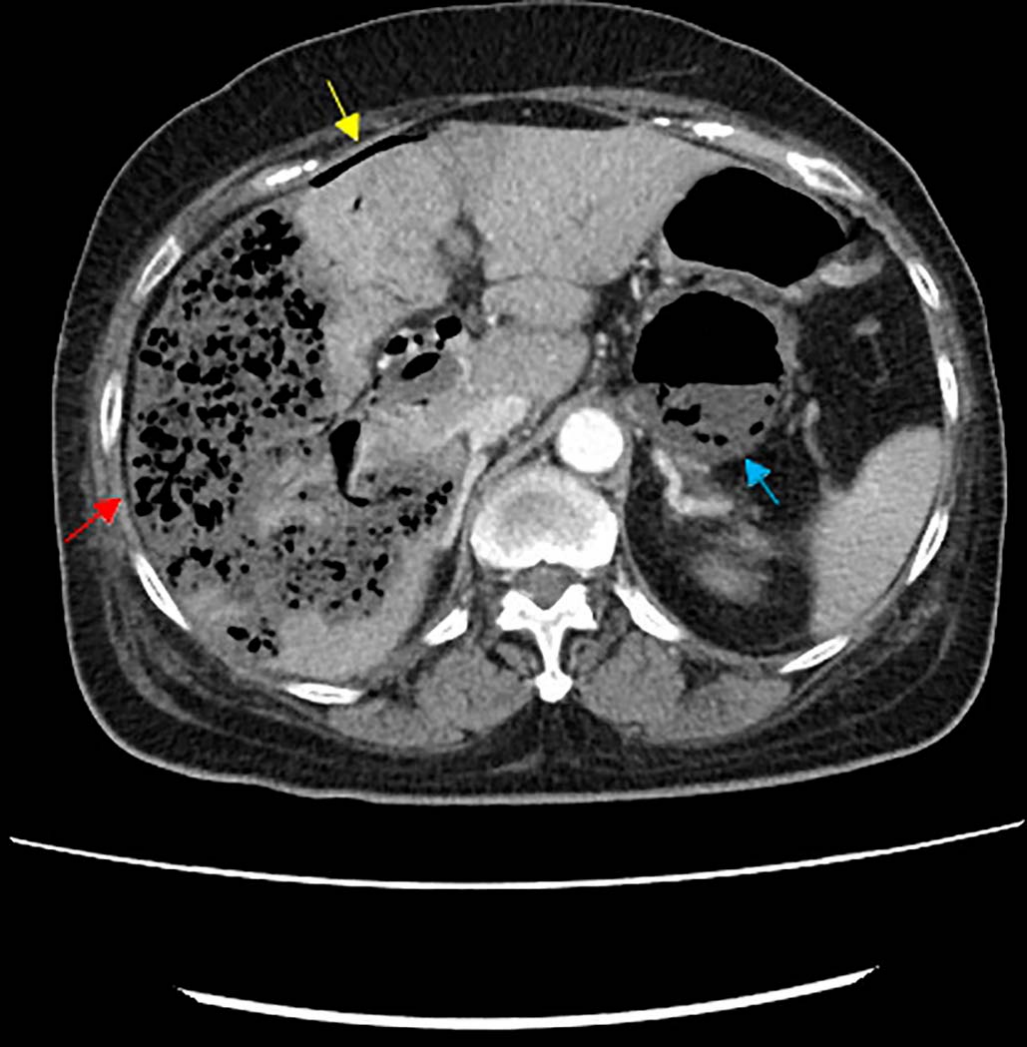
Abdominal computed tomography demonstrated multifoci of liver necrosis with gas-forming abscesses (red arrow), pneumoperitoneum (yellow arrow), and retroperitoneal abscesses (blue arrow).

The first roentgenogram showed a right-side elevated diaphragm with ileus only. Unfortunately, the patient deteriorated within 2 hours. Profound septic shock developed. The bedside abdominal radiography demonstrated gas accumulated in the right subphrenic space (Figure 1). Abdominal computed tomography revealed multifoci of liver necrosis with gas-forming abscesses, pneumoperitoneum, and retroperitoneal abscesses (Figure 2).

Emergent surgery with necrosectomy and drainages were performed on both liver and retroperitoneal abscesses. An emphysematous liver abscess was diagnosed. Blood cultures and pus cultures ultimately all yielded *Klebsiella pneumonia.* Poorly controlled hyperglycemia is believed as an important risk factor for this rapidly progressive condition. The patient eventually died within 24 hours after admission.

Emphysematous liver abscesses are a critical condition. The estimated epidemiology among all bacterial liver abscesses is 6%–24% and higher among diabetic patients. The fatality rate is extremely high at 27%, necessitating prompt intensive care. The most frequently isolated organisms are polymicrobial. *Klebsiella pneumonia* accounts for more than 70% in Taiwan. Ultrasound and abdominal computed tomography should be performed early in clinical suspicious cases. Among the critical patients, invasive interventions such as percutaneous drainage, broad-spectrum antibiotics, and optimal glucose control are the main treatments. Surgery should be performed if the abscess ruptures with peritonitis.^1,2^

## DISCLOSURES

Author contributions: All authors contributed equally to this manuscript. Y-C Tsai is the article guarantor.

Financial disclosure: None to report.

Informed consent was obtained for this case report.

## References

[1] Takano Y Hayashi M Niiya F. Life-threatening emphysematous liver abscess associated with poorly controlled diabetes mellitus: A case report. BMC Res Notes. 2017;10:117. 28264703 10.1186/s13104-017-2445-8 PMC5340034

[2] Chou FF Sheen-Chen SM Chen YS. The comparison of clinical course and results of treatment between gas-forming and non-gasforming pyogenic liver abscess. Arch Surg. 1995;130:401–5. 7710340 10.1001/archsurg.1995.01430040063012

